# Gold Nanoparticle Enabled Localized Surface Plasmon Resonance on Unique Gold Nanomushroom Structures for On-Chip CRISPR-Cas13a Sensing

**DOI:** 10.1002/admi.202201261

**Published:** 2022-11-27

**Authors:** Jacob Waitkus, Yu Chang, Li Liu, Srinivasu Valagerahally Puttaswamy, Taerin Chung, Adrian M. Molina Vargas, Stephen J. Dollery, Mitchell R. O’Connell, Haogang Cai, Gregory J. Tobin, Nikhil Bhalla, Ke Du

**Affiliations:** University of California, Riverside, Riverside, CA, USA; University of California, Riverside, Riverside, CA, USA; University of California, Riverside, Riverside, CA, USA; NIBEC School of Engineering, Ulster University, Belfast, UK; Healthcare Technology Hub, School of Engineering, Ulster University, Belfast, UK; Tech4Health Institute and Department of Radiology, New York University Langone Health New York, USA; University of Rochester Medical Center, University of Rochester Rochester, USA; Biological Mimetics Inc, Frederick, MD, USA; University of Rochester Medical Center, University of Rochester Rochester, USA; Tech4Health Institute and Department of Radiology, New York University Langone Health New York, USA; Biological Mimetics Inc, Frederick, MD, USA; NIBEC School of Engineering, Ulster University, Belfast, UK; Healthcare Technology Hub, School of Engineering, Ulster University, Belfast, UK; University of California, Riverside, Riverside, CA, USA

**Keywords:** CRISPR-Cas13a, finite difference time domain, gold nanomushroom, gold nanoparticle, localized surface plasmon resonance (LSPR), microfluidics, SARS-CoV-2

## Abstract

A novel localized surface plasmon resonance (LSPR) system based on the coupling of gold nanomushrooms (AuNMs) and gold nanoparticles (AuNPs) is developed to enable a significant plasmonic resonant shift. The AuNP size, surface chemistry, and concentration are characterized to maximize the LSPR effect. A 31 nm redshift is achieved when the AuNMs are saturated by the AuNPs. This giant redshift also increases the full width of the spectrum and is explained by the 3D finite-difference time-domain (FDTD) calculation. In addition, this LSPR substrate is packaged in a microfluidic cell and integrated with a CRISPR-Cas13a RNA detection assay for the detection of the SARS-CoV-2 RNA targets. Once activated by the target, the AuNPs are cleaved from linker probes and randomly deposited on the AuNM substrate, demonstrating a large redshift. The novel LSPR chip using AuNP as an indicator is simple, specific, isothermal, and label-free; and thus, provides a new opportunity to achieve the next generation multiplexing and sensitive molecular diagnostic system.

## Graphical Abstract

A nanoplasmonic system based on the coupling of gold nanomushrooms and gold nanoparticles is introduced, showing a giant plasmonic resonant shift up to 31 nm. The gold nanoparticles labeled by CRISPR-Cas13a assay are non-specifically deposited on the gold nanomushrooms through a microfluidic cell for the accurate recognition of target viral RNA.

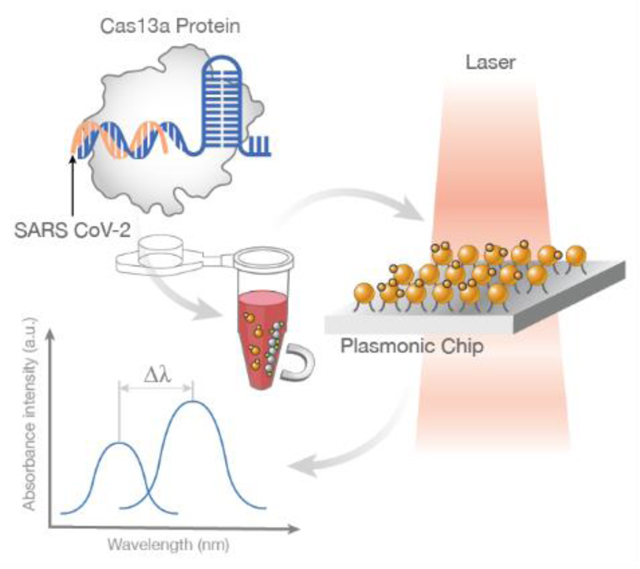

## 1. Introduction

Surface plasmon resonance (SPR) results from the coupling between free electrons and photons, where combined electromagnetic oscillations are utilized to enhance the light–matter interactions.^[1]^ In other words, a characteristic intensity spectrum with a shifting peak resonance wavelength value is achieved due to changes in the local refractive index. The free electrons that exist in various metallic nanostructures, with a subwavelength thickness, play an essential role and have been used widely for the characterization of biomolecular interactions.^[2]^ Localized surface plasmon resonance (LSPR), on the other hand, restricts the coupled electromagnetic oscillations of SPR to a local area, resulting in a sharper spectrum of the local resonance peak.^[3]^ In addition, the performance of LSPR systems in producing an absorbance spectrum is significant and usually independent of the incident angle unlike SPR devices, enabling numerous applications such as molecular sensing,^[4]^ reaction monitoring,^[5]^ and biomedical imaging.^[6]^

We previously pioneered a novel LSPR sensing platform based on randomized gold nanomushrooms (GNMs) on a SiO_2_ substrate, achieved by a simple gold film deposition, one-step annealing, and reactive ion etching (RIE) process.^[7]^ This LSPR-based GNM chip was then applied for real-time and label-free monitoring of *Escherichia coli* biofilms,^[8]^ sensing of multiple viral variants, and screening of protein kinase activity.^[10]^ LSPR can also be used for photothermal heating; thus, enabling on-chip polymerase chain reaction (PCR) for DNA sensing.^[11]^ Recently, LSPR has also been integrated with emerging CRISPR-Cas12a assays to enable the detection of genetic modifications in crops by the naked eye. In this application, nonspecific collateral cleavage by CRISPR-Cas12a induces an invertase-glucose oxidase cascade reaction for sensitive and simple signal readout.^[12]^

Here, we introduce a novel and ultrasensitive nanoplasmonic sensing platform that utilizes the formation of AuNM and streptavidin-coated gold nanoparticles (AuNPs) clusters to enable LSPR coupling. The randomly distributed clusters interact with the GNMs, leading to a resonant wavelength shift of ≈14 nm with the coated 40 nm AuNPs at concentrations as low as 100 pm. In addition, the resonant shift is correlated with the GNP concentration and a ≈31 nm redshift is recorded with 500 pm input, which is well explained by our simulation results. Using GNPs as an indicator, we then show that this novel LSPR chip can be coupled with a CRISPR-Cas 13a RNA detection assay for the specific identification of SARSCoV-2 RNA through target recognition, collateral cleavage, and AuNM–AuNP coupling. Our platform relies on a rapid maskless fabrication process, does not require complicated surface modification, and can be interpreted by a simple optical setup; thus, establishing a powerful method for multiplexing, isothermal, and sensitive biosensing applications.

## 2. Results

**Figure 1.**
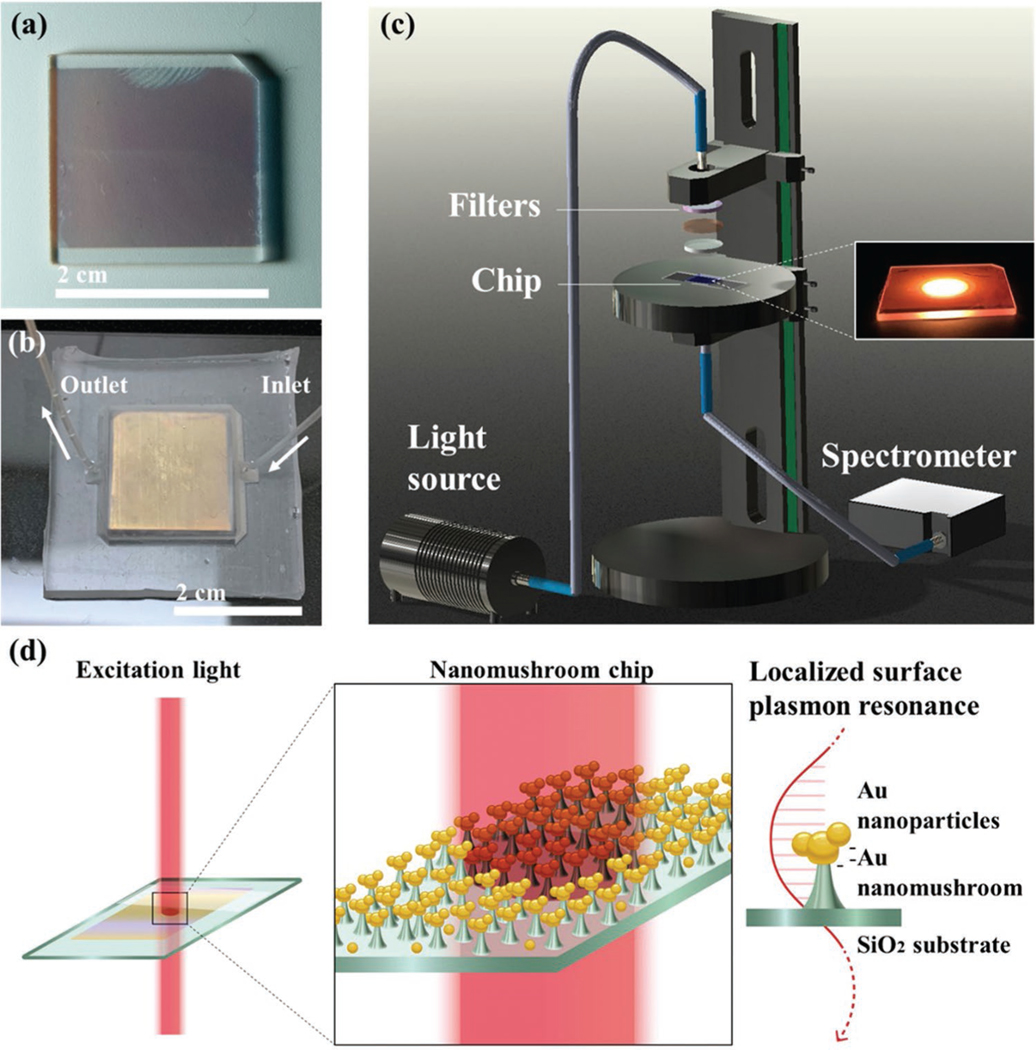
a) AuNM chip prepared by the one-step RIE etching process. b) PDMS-based microfluidic chamber for the sealing of AuNM chip. c) Setup of the absorbance measurement. The inset shows the beam spot during excitation. d) The schematic of the LSPR between AuNPs and AuNMs.

**Figure 2.**
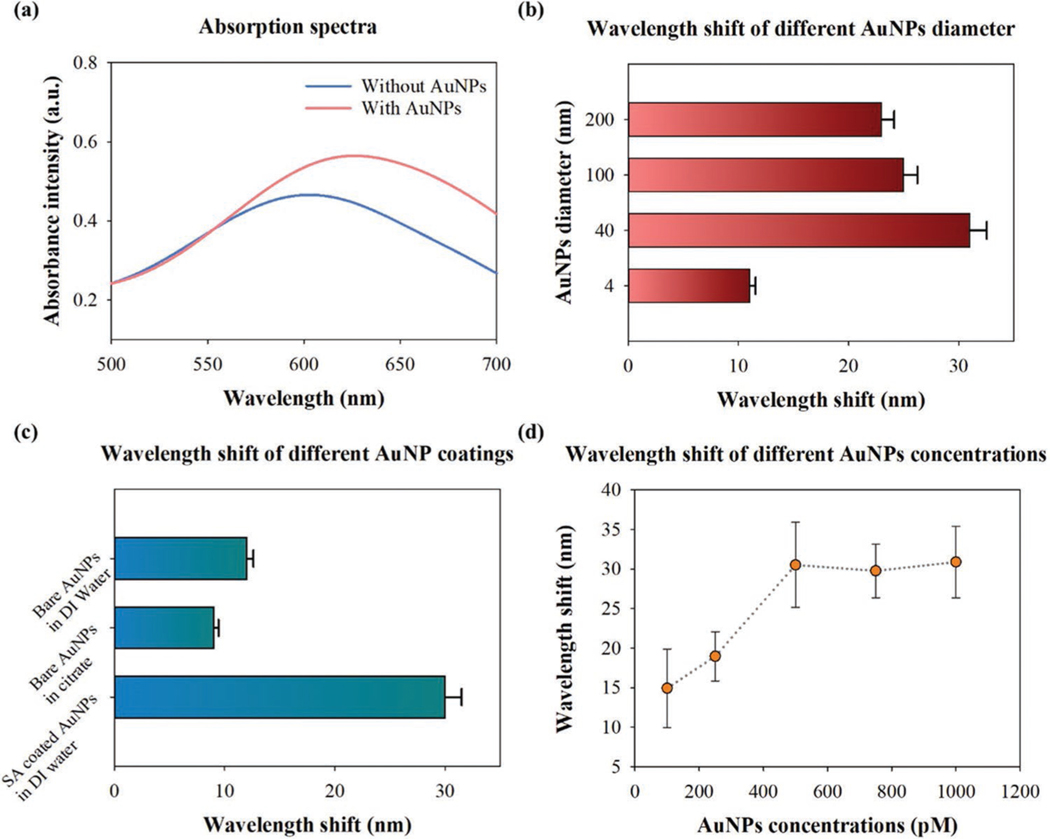
a) Absorption spectrum of AuNMs before (blue) and after (red) the coating of 40 nm AuNPs. b) Wavelength shift of the AuNPs (1.1 nm) coated AuNMs with a diameter ranging from 4 to 200 nm (one-way ANOVA MATLAB R2019b, *n* = 3, *P* = 1.32e-06). c) The wavelength shift of different AuNPs coatings (1.1 nm, 40 nm) (one-way ANOVA MATLAB R2019b, *n* = 3, *P* = 1.22e-05). Bare AuNPs were prepared in DI water and citrate solutions, respectively, whereas the streptavidin-coated AuNPs were suspended in DI water. d) Wavelength shift on GNM substrate versus 40 nm AuNPs with different concentration (one-way ANOVA MATLAB R2019b, *n* = 3, *P* = 0.0005).

As shown in Figure 1a–d, the LSPR interactions between AuNMs and AuNPs changed the local refractive index at the interface and resulted in a redshift in the absorption spectra. Figure 2a demonstrates the absorption spectra of the AuNMs with and without AuNPs. An increased absorbance intensity from 0.46 to 0.56 and a redshift from 600 to 624 were observed in the samples where AuNPs (1.1 nm) were added to the surface. Next, we measured the wavelength shift of AuNPs with different diameters ranging from 4 to 200 nm by fixing the concentration at 1.1 nm. As shown in Figure 2b, AuNPs with a diameter of 40 nm showed the largest redshift in the peak wavelength (≈30 nm). In contrast, only an ≈11 nm redshift was observed for 4 nm AuNPs. We then studied how surface coating affects the binding of 40 nm size AuNP on AuNMs by using 40 nm nanoparticles (Figure 2c). Streptavidin-coated AuNPs exhibit the largest wavelength shift (≈30 nm) among the groups, whereas the samples with uncoated AuNPs show a much smaller wavelength shift regardless of solution (DI Water/ Citrate). We then extend the use of the chosen AuNP (40 nm) to optimize the concentration of AuNP solution for maximizing the wavelength shifts, required for coupling it with AuNM. The wavelength shift versus AuNP concentration is depicted in Figure 2d. Approximately 15 and 20 nm redshift were detected for 100 and 250 pm AuNPs, respectively. At concentrations greater than or equal to 500 pm, the wavelength shift observed appears stable at ≈31 nm. This may be due to the localized aggregation of AuNPs reaching a maximum quantity for the limited space surround a AuNM structure.

**Figure 3.**
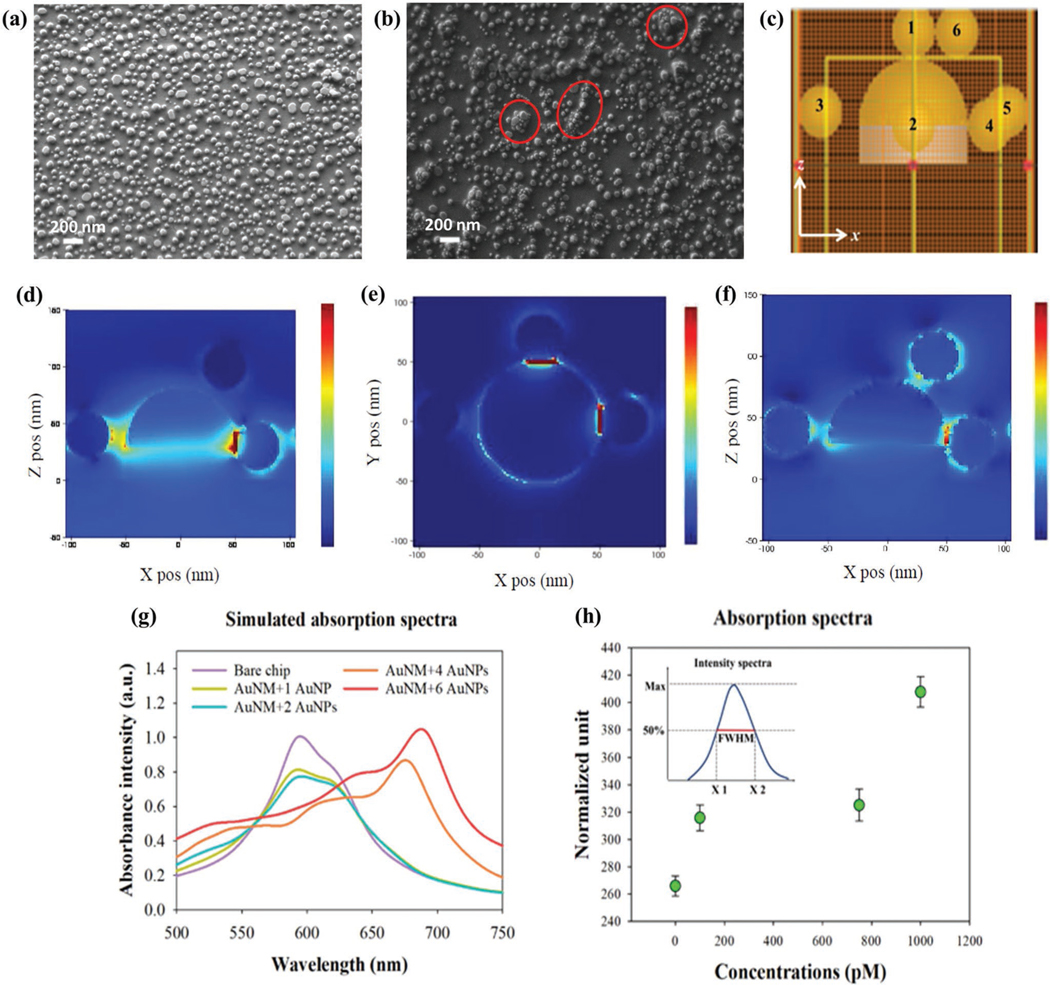
The SEM image of the a) bare and b) AuNP-coated AuNM substrate. c) The 3D numerical modeling environment of single AuNM with six AuNPs. As a light source, incident plane wave is excited along the *z*-axis. The numbers denote the sequence of randomized AuNPs added onto the AuNM. The calculated electric field distributions in the d) *xz* plane (polarization 45°), e) *xz* plane (max. wavelength shift), and f) *xz* plane (polarization 45°). g) 3D FDTD simulated spectra of a single AuNM with different numbers of randomized AuNPs. h) Normalized absorption spectra data of different AuNPs concentrations. The normalized unit was calculated based on FWHM divided by the peak intensity of the experimental spectra for different concentrations of the AuNPs.

SEM images of the AuNM chip with and without the addition of 40 nm AuNPs are shown in Figures 3a and 3b, respectively. The randomly localized aggregation of AuNPs on the AuNMs, marked by red circles, would contribute a larger redshift than a single AuNP or bare AuNM. The random aggregation of the GNPs was shown to be produced with high consistency by statistical analysis in MATLAB R2019b. The absorption measurements were performed across a 2 × 2 cm chip by measuring a spot 2.50 mm in diameter. This ensured that the three measurements taken were at entirely different points. Comparing all measured spots, the variation between peak values was less than 5 nm at the extremes. This difference was within the noise of the system, highlighting the consistency and repeatability of the device for the formation of the GNP-AuNM clusters. We performed a 3D finite-difference time-domain (FDTD) simulation to verify the experimental results. Several AuNPs were added onto a AuNM randomly, as shown in Figure 3c. The sizes of the AuNPs and the AuNM were fixed at 40 and 100 nm, respectively. The total field scattered field was assumed to be the input from the top of the structures. Figure 3d,e depicts the *yz* plane-cross sectional distribution in 45° polarization and *x*-axis polarization. The latter shows a maximum wavelength shift to 631 nm, whereas the former shows only 623 nm, which is similar to the *xz* plane-cross sectional distribution with 45° polarization (Figure 3f). This result shows that the relative positions of AuNPs in the measured plane would result in different wavelength shifts.

The peak wavelength with different numbers of 40 nm AuNPs on AuNMs is demonstrated in Figure 3g. The smallest wave-length shift is observed when a single AuNP is present on the AuNM. When the number of particles increases, a more significant wavelength shift results. Furthermore, the spectral shape undergoes broadening with the increase of AuNPs. In general, regardless of the number of AuNPs, the positions and distance to AuNM with regard to the polarization state of light determine the transmission wavelength and the degree of spectral broadening.^[13]^ This could be verified by our normalized experimental spectra presented in Figure 3h. The normalization of each spectrum was calculated based on the full width at half maximum (FWHM) divided by the peak intensity. The data of the blank sample (without AuNPs) shows the lowest level. Interestingly, 100 and 750 pm samples show a similar level while the concentrations are different, suggesting the ratios of their FWHM and peak intensity are close. The 1000 pm sample shows the highest FWHM level, indicating that the resultant ratio of the broadened spectrum and the peak intensity are substantially larger. This result agrees with our simulation data as the resultant bandwidth of 1, 2, and 4 AuNPs are different even with similar peak intensities (Figure 3g). It should be noted that the relative positions between the AuNPs and AuNMs may affect the resultant intensity even though the number of particles is the same.

**Figure 4.**
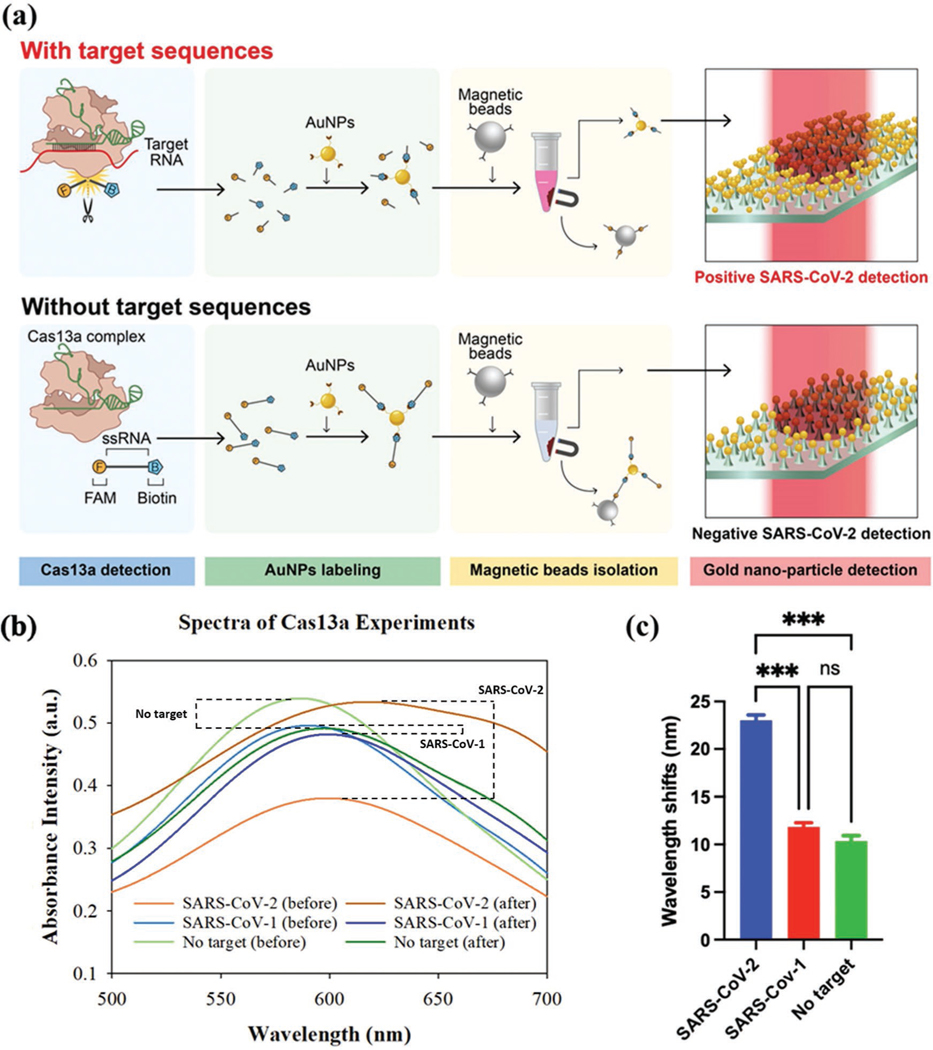
a) Schematic of CRISPR-Cas 13a based RNA sensing with AuNP–AuNM interaction. In the presence of the target RNA (red), the fluorescently labeled ssRNA reporters are cleaved and left in the supernatant. b) Measured wavelength shift for positive (SARS-CoV-2), negative (SARS-CoV-1), and blank (no target) samples. The target concentration was 100 nm. c) Summarized wavelength shift for the positive, negative, and blank samples.

We next show that the AuNP–AuNM platform can be integrated with a CRISPR-Cas13a assay for nucleic acid sensing.^[14]^ As shown in Figure 4a, when the Cas13a complex is activated by a guide RNA (green) and a target RNA (red), the fluorescently labeled ssRNA reporters are cleaved and this prevents the conjugation of streptavidin-coated AuNPs and anti-fluorescein antibody-coated magnetic beads. As a result, the isolated AuNPs remain in the supernatant. This addition allows for the formation of the AuNP–AuNM couple on the surface of the chip. On the other hand, without target RNA, the ssRNA reporters are not cleaved by the Cas13a, and the AuNPs are captured and isolated by the magnetic beads. As a result, almost no AuNPs can be coupled to the AuNM substrate. The SARS-CoV-2 RNA is not directly measured on the surface of the AuNM substrate. It is instead detected through the identification of the large-scale redshift caused by the addition of GNPs to the plasmonic system. The GNPs exist in the supernatant solution after magnetic bead isolation. It is also worth mentioning that no magnetic beads are present in the supernatant as they are attracted to the magnet on the side wall of the target vial. As shown in Figure 4b, for positive samples, the isolated AuNPs are deposited on the AuNM sample; thus, changing the localized refractive index and leading to a large redshift. On the other hand, only an ≈10 nm redshift is observed for either the negative or blank sample, indicating the high specificity of our method. The summarized wavelength shift for the 100 nm target is shown in Figure 4c, clearly showing the difference between positive and negative samples. The concentration used here is far above common detection methods for the SARS-CoV-2 virus. In this work, it was used as a model pathogen for a proof-of-concept of the LSPR device. Due to the amplification increase reported for loop-mediated isothermal amplification (LAMP) and PCR techniques,^[15–19]^ it would be possible for identifying concentrations of the SARS-CoV-2 as low as 100 am to 1 fm, given the 100 nm detection reported here. This concentration is within the range identified as the required viral load for SARSCoV-2 detection.^[20,21]^

## 3. Discussion

To form GNP and GNM couplings, we introduced a simple direct addition protocol. This physical adsorption protocol is faster than other surface binding techniques such as thiol binding,^[22,23]^ amine coupling,^[24,25]^ or salt aging.^[26,27]^ Upon addition of the GNPs to the substrate, a pseudo-aggregation of the GNPs onto the GNMs occurs due to the charge density of the GNPs. The streptavidin-coated GNPs (40 nm) are negatively charged which bind to the negative GNM and other GNPs to form stable agglomerates. This is desirable because the clusters disperse the surface charge and minimize the localized energy on the surface which is favored by the nanoparticles.^[28]^ The detection of the GNPs was categorized by the magnitude of the resonant spectrum peak wavelength shift. Changes in intensity between spectra before and after the addition of these particles was not considered a factor due to the inconsistencies associated with the AuNM substrate, as well as the measurements.^[7,29,30]^ The AuNM layer had inherent differences from other samples due to changes in the SiO_2_ layer. Although in some instances a large increase was observed in peak intensity, this was not directly correlated with the presence of GNPs for this system in all cases. Due to these factors, wavelength shift is the common parameter used to characterize LSPR devices.^[31]^ For our measurements, we relied on the wavelength shift observed exclusively. Through adding surfactants or other charge neutralizing coatings (such as citrate molecules) to the nanoparticles, their ability to aggregate decreases due to the lessened surface charge which lowers the efficiency of the direct addition protocol. Other than surface chemistry, the size of the GNP and GNM also play an important role in the formation of the aggregates. As the diameter of the GNP decreases, the ratio of total surface area to volume increases, resulting in a more intense negative surface charge per unit area that is favorable for metallic bonding.^[32]^ The decreasing diameter also provides more spaces for the surrounding GNPs to fill. This helps to supplement the formation of larger aggregates of the nanoparticles onto the GNMs.

The redshift observed in the adsorption spectra is caused by the change in refractive index of the GNM substrate. Through the addition of GNPs to the surface, the peak wavelength value of the spectra is shifted to higher wavelengths. The size of this shift is contingent on numerous factors including the diameter of both GNM and GNP, the surface condition of the GNPs, and the concentration of the GNPs themselves. For this work, the size of the GNMs varies from 100 to 200 nm and the diameter of the GNPs we tested ranged from 4 to 200 nm. The largest redshift observed was recorded for the 40 nm GNPs with a shift of ≈30 nm. At smaller diameter, the GNP exhibits LSPR damping which lessens the enhancement of the incoming photons to produce a less intense and wider spectrum.^[33]^ The redshift is also decreased as the GNPs have less of an effect on the surrounding environment and are much smaller than the wavelength of the incident light. Larger GNPs demonstrate greater LSPR shifts as a result of stronger scattering of the photons in “hot spots” or areas with a greatly enhanced electric field.^[34]^ We found that GNPs larger than 100 nm failed to produce a redshift greater than that of the 40 nm GNPs. These larger GNPs favor aggregation with other GNPs as opposed to the substrate, resulting in them being unbound to the surface and removed in the washing process.

The effect of the molecular coating on LSPR shift was also investigated. The bare GNPs result in a reduced redshift due to the presence of charge neutralizing citrate molecules.^[35]^ For GNPs of identical diameter, the bare GNPs only produce a 10 nm redshift as opposed to the 30 nm redshift exhibited by the streptavidin-coated GNPs. Thus, we select streptavidincoated NPs as our biosensor indicator. We show that the observed LSPR resonance shift is correlated to the GNP concentration, and it can reach a critical value when the GNPs saturate the GNMs. The hot spot intensity grows stronger until there is little space left for additional GNPs. The increasing quantity of GNPs also results in a broadening of the absorption spectra. This effect can also be observed for larger diameter GNPs. This is caused by the formation of a mixed-size distribution of GNPs. Within these large clusters, the GNPs exhibit higher order oscillations due to the particles resonating in-phase and out-of-phase with one another. This is similar for larger diameter GNPs where the electrons can oscillate through a greater space.

We used 3D-FDTD simulations to explain the giant resonant shift and show that by adding more GNPs the redshift is increased. However, the experimentally gathered wavelength shifts are smaller than the theoretical value. This can be explained by the presence of PBS solution and DI water which alter the refractive index of the surface. In addition, redshifts resulting from the simulations exhibit sharper peaks when compared to the experimental values. This is caused by the simplified conditions used in the simulation, whereas experimentally, there were arrays of GNMs rather than single GNM. The resultant spectrum should be a normalized distribution of the combination of GNMs with and without the binding of GNPs.

We also use a plasmonic ruler system to calculate the resonant shift. Given a known wavelength shift due to the particles binding, the distance between can be calculated,^[36,37]^

(1) $$\frac{\text{Δ}\lambda }{\lambda_{0}}=0.12\exp\left\{\frac{\left(-\text{s}/D\right)}{0.16}\right\}$$

Where *Δλ* is the difference in peak resonant wavelength value after the coupling, *λ*_o_ is the initial apparent wavelength of the AuNM substrate, *s* is the interparticle separation distance between AuNP and AuNM, and *D* is the diameter of the AuNPs used, not including the streptavidin coating. This was done because the streptavidin is being calculated as the separation distance.

For this work, the equation was modeled for an AuNP–AuNP binding event. Although the AuNMs are a constructed substrate, their unique geometry allows for them to act as an AuNP due to their exposed surface. For this equation, few variables are required including an initial particle diameter, set at 40 nm for the streptavidin-coated AuNPs, and the apparent wavelength peak of the AuNM before binding which was designated as 580 nm. The thickness of the streptavidin layer was ignored for this calculation as the thickness of the coating was being investigated for the interparticle separation. Other necessary components needed were the universal trend constant to be set at 0.12 and the decay constant for AuNP interactions to be 0.16.^[36]^ The result of a 30 nm wavelength shift demonstrates that a 5.39 nm distance would be required to separate the nanoparticles (Figure S1, Supporting Information). This directly corresponds to the thickness of the streptavidin coating on the AuNP.^[38]^

The redshift of our GNP-GNM system shows greater sensitivity than most of the LSPR systems and is ideal for biosensing applications. In addition, the non-specific binding of the GNPs is simple without relying on complicated surface treatment. We show that by combining with a CRISPR-Cas13a assay, our system can be used for viral RNA detection. Biosensors based on LSPR have numerous advantages such as being label-free, sensitive, and highly multiplexable. The detection scheme relies on absorption spectrum without using dye labels. Thus, a simple spectrophotometer based on an inexpensive white light source is sufficient for detection. LSPR chips with nanostructures were previously fabricated via complicated processes such as nanolithography, lift-off, or metal etching.^[39,40]^ In our work, the GNMs were created by a one-step RIE etching process, which shows excellent long-range order and is more suitable for low cost and scalable production. In the future, our chip can be segmented to many individual cells for multiplexed sensing. To further enhance the detection sensitivity of target RNA, our system can work with a conventional PCR^[41]^ or other types of isothermal amplification methods^[42,43]^ before the on-chip CRISPR-Cas reaction. The system developed here offers the unique advantages resulting from the coupling of the GNPs to the AuNM structures for the first time. Combining both components produces the giant plasmonic resonant shift reported. This combats the bottleneck that often afflicts plasmonic systems, where low limits of detection are hard to achieve.^[44–46]^ The device fabricated here offers rapid, label-free detection at a highly sensitive level when compared to current plasmonic systems.

## 4. Experimental Section

### Fabrication of AuNMs:

The fabrication process for AuNMs was reported by the authors previously.^[7]^ Briefly, a SiO_2_ substrate was cleaned by acetone and isopropanol before the following deposition of a 8 nm Au film. The deposition was implemented in an e-beam evaporator at a rate of 0.1−0.2 Å s^−1^. Subsequently, the Au thin film was annealed at 560 °C for 3 h. The annealing transformed the Au film into nanoislands which then became anchored into the SiO_2_ layer. The previous annealing step was crucial for anchoring the nanoislands that formed into the SiO_2_ substrate. This prevented their lift-off in the accompanying RIE etch. The chip was then sent to a 5 min RIE process, performed by inductively coupled plasma (ICP) chemical vapor deposition (CVD) equipment (Plasmalab 100, Oxford Instruments), to realize selective etching for generating the unique AuNM structures as shown in Figure 1a. This etch was done with a SF_6_ plasma at 5 °C. The end product resulted in a random array of AuNM structures across the glass substrate. These final structures had a diameter of 100 nm for the nanoisland and a height of 30–40 nm for the SiO_2_ pillar. The structures showed high uniformity in absorbance peak as the entire chip was within 2 nm for the peak wavelength value. The fabrication process is shown in Figure S2, Supporting Information.

### Fabrication of the Microfluidic Chip:

The AuNMs were sealed in a PDMS microfluidic chamber for surface treatment. Silicon elastomer base and curing agent (SYLGARD 184) were mixed in a 10:1 ratio and poured over a 3D printed resin mold in a glass petri dish. The resin mold was manufactured via 3D printing (Form2, Formlabs) at a size of 2.2 × 2.2 × 4 cm to adequately contain the AuNM chip, which was 2 × 2 cm in area, as well as allow room above the substrate for fluid flow. The PDMS mixture was then placed under vacuum for 30 min to remove any air bubbles and baked in an oven at 75 °C for ≈5 h. Following the bake, the PDMS chamber was removed from the resin mold and cleaned in an ultrasonic bath. Two 1 mm holes were punched in opposite corners of the PDMS chamber to form the inlet and outlet of the system as shown in Figure 1b. A 2 × 2 cm portion of double-sided tape was cut and used to adhere the AuNM chip to the surface of a treated glass slide, careful that the topside of the substrate did not come into contact with anything. Finally, the PDMS layer was placed around the chip and adhered to the glass slide on a hot plate at 125 °C overnight. This resulted in a liquid-tight chamber of ≈240 μL to hold the test samples and AuNM substrate.

### AuNP–AuNM Interaction:

The Streptavidin-coated AuNPs (40 nm) were purchased from nanoComposix Inc. The bare (citrate-coated) AuNPs (4–200 nm) with concentrations ranging from 0.115 nm to 9.26 μm were adjusted to 1.1 nM through dilutions with UltraPure Distilled Water (Life Technologies). The 4, 100, and 200 nm nanoparticles were purchased from Luna Nanotech and suspended in water with 0.01% Tween-20 while the bare 40 nm nanoparticles were acquired from nanoComposix Inc. in an aqueous 0.02 mm sodium citrate solvent. For exchanging the carrier of the bare 40 nm AuNPs to DI water from the citrate solution, a sample of 10 μL was centrifuged at 1500 × *g* for 45 min. The supernatant was then extracted and replaced with an identical quantity of DI water. This solution was vortexed to aid in the resuspension of the AuNPs. The same total volume was used for each sample once identical concentrations were achieved. These samples were then diluted with DI water to a total volume of 240 μL for later application to the AuNM surface. The AuNPs (240 μL) were added to the PDMS microfluidic chamber and incubated on the surface of the AuNMs for ≈12 h at room temperature. Following incubation, the surface of the substrate was then washed repeatedly using 730 μL of 1× PBS buffer. The washing flow rate was controlled to ensure that minimal AuNPs would be dislodged from the surface of the chip. This was performed with a SP2201 Syringe Pump (World Precision Instruments). After washing, the AuNM substrate was extracted from the microfluidic device and allowed to air dry overnight at room temperature in a covered petri dish. This was to ensure that no solution remained on the surface and no particulate settled on the surface during drying that would influence the resulting absorbance spectrum of the chip.

### CRISPR-Cas13a Experiments:

The Lbu-Cas13a protein was prepared based on our established protocols and described in our previous publications.^[47]^ The target SARS-CoV-2 (703 nucleotides) and negative control SARS-CoV-1 (660 nucleotides) spike gene fragments (sequences are listed in Table S1, Supporting Information) were synthesized and cloned into plasmids pUC57-SARS-CoV-1 and pUC57-SARS-CoV-2. The fragments were amplified via PCR and TA cloned into vector TOPO-TA directly following a T7 promotor (Invitrogen, MA, USA). To generate linear template DNA for RNA synthesis, plasmids were digested at the 3′ terminus of the Spike coding fragments with HindIII (New England Biolabs, MA, USA), EtOH precipitated and then re-suspended in dH_2_O. RNA fragments were synthesized using linear plasmid DNA as a template via T7 runoff reactions incubated for 4 h at 37 °C (RiboMAX, PROMEGA, WI, USA). Following RNA synthesis, reactions were DNase treated as per the manufacturer’s instructions. Proteins and excess nucleotides were removed by silica gel membrane column purification (RNeasy- QIAGEN, MD, USA). RNA size and initial quantitation were performed via agarose–TBE gel electrophoresis with RiboRuler RNA ladder (ThermoFisher, MD, USA) using densitometry. Quantitation was verified via UV absorption spectroscopy at 260 nm. Guide RNA and RNA reporters were purchased from IDT Inc. (Table S1, Supporting Information). Dynabeads MyOne Streptavidin C1 with a diameter of 1 μm was purchased from Thermal Fisher Scientific Inc. Streptavidincoated AuNPs with a diameter of 40 nm (1.1 nm) were purchased from nanoComposix Inc. Biotinylated anti-fluorescein antibodies (1 mg mL^−1^) were purchased from Vector Laboratories Inc. The AuNP-based CRISPR Lbu-Cas13a reaction was carried out according to the authors’ previous work.^[48]^ The Cas13a-RNA reporter probe mixture contained 8 μL CRISPR complex (100 nm Lbu-Cas13a, 110 nm crRNA, and 1× STD buffer), 4 μL of the biotin–fluorescein ssRNA reporter (10 μm), 8 μL RNA target (100 nm), 16 μL 5× STD buffer, and 44 μL Nuclease Free Water. The reaction was incubated at 37 °C for 30 min. To immobilize RNA reporters onto streptavidin-coated AuNPs, 40 μL of concentrated AuNPs (5.5 nm) were added to 80 μL of Cas13a-RNA reporter probe mixture with different target RNAs. The samples were incubated on a rotary mixer at room temperature for 15 min. To isolate noncleaved ssRNA-AuNP reporters, Streptavidin-coated magnetic beads were washed three times with 1 × PBS buffer. Forty microliters of biotinylated anti-fluorescein antibody (1 mg mL^−1^) were added to the 40 μL Dynabeads solution, followed by incubation at room temperature for 30 min. After incubation, the beads were washed three times with 1× PBS buffer to remove any unbound anti-fluorescein antibodies. Next, 120 μL AuNP-labeled Cas13a reaction products from the last step were added to the washed magnetic beads for 30 min. After the reaction, the magnetic beads were isolated by a magnet and the supernatant was left for the on-chip testing.

### Absorption Measurement:

The spectrophotometer was built as shown in Figure 1c, consisting of a halogen light source (Ocean Optics, 085510801), a set of filters, a transmission stage (Ocean optics, STAGE-RTL-T), and a spectrometer (Ocean Optics, FLMS16493). The set of filters included a UV–vis collimating lens (Ocean optics), broadband dielectric mirror (Thorlabs, BB1-E02), and a short-pass filter (Thorlabs, FESH0750). A glass substrate was placed on the movable sample holder plate to hold the AuNM chip. The spectrum was collected from three randomly chosen points on the chip surface for each measurement. The setup measured a circular spot on the surface with a diameter of 2.50 mm. The integration time was set at ≈0.2 s, and three scans were averaged for every measured point. The wavelength shift was defined as the difference of average peak wavelength from measurements taken before and after the experiment.

### Simulation:

The 3D FDTD modeling was performed for a single AuNM and multiple AuNPs to numerically verify the experimental results with a commercial software package (Lumerical FDTD solutions 2021 R2). The size of the AuNPs and the AuNM were fixed at 40 and 100 nm, respectively, using the dielectric permittivity model.^[35]^ For modeling, the incident plane wave light source was employed from the top of the structures. The spectra resulting from the random addition of AuNPs onto the AuNM were collected from the far-field monitoring domain. Furthermore, the cross-sectional electrical field distributions in terms of different polarizations were calculated for visualizing plasmonic hot spots from the LSPR effect due to the number of AuNPs and the relative positions between them and the AuNM.

### Statistical Analysis:

For the preparation of the figures, minimal preprocessing of the data was performed. In Figure 3h, the *y*-axis incorporated a normalized unit to best characterize the broadening of the absorption spectra at various AuNP concentrations. The normalization was performed using the full width distance of the specific spectrum at half the peak intensity. No other data and figures underwent pre-processing. For each figure, the spectra, data points, and columns were representative of the mean value across the surface of the chip. The mean value was taken from three separate points on the surface of the 2 × 2 cm AuNM substrate. The error bars shown in the figures represent the standard deviation (SD) of the average and were set at 5% of that value. This was to account for variations between the data points that wasn’t represented by the average. For assessing the statistical significance between the various experiments, MATLAB R2019b was used to perform one-way ANOVA testing on the effects of diameter and coating on the AuNP and the difference among positive, negative, and control samples for the SARS-CoV-2 experiment. The statistical analysis used *n* = 3 for sample size and *P* < 0.05 to be a significant statistical difference. Assumptions within the ANOVA test require that the samples are normally distributed and independent of one another, which is valid for this data set.

For the diameter comparison, a *P* value of 1.32e-06 was produced. It was shown that the 30 nm shift recorded for the 40 nm AuNPs was significantly different from the results of the 4, 100, and 200 nm particles. The respective table and box plot can be found in Figure S3 and Table S2, Supporting Information. The *P* value for the coating experiment was 1.22e-05, indicating that the difference in the peak values was significant. These results, shown in the Supporting Information, highlight the statistical difference between the streptavidin-coated and bare AuNPs. The analysis for the two bare (citrate-coated) samples was shown to have a strong overlap in their results, indicating their lack of statistical difference. The final analysis was performed on the CRISPRCas13a results, producing a *P* value equal to 0.0005. The SARS-CoV-1 and no target sample produced significantly similar results with no real differences. Both of these data sets were vastly different from that of the SARS-CoV-2 sample set, as demonstrated by the plots listed in the Supporting Information. Therefore, the CRISPR-LSPR combination allows for sensitive detection over negative samples and control experiments for the positive identification of the SARS-CoV-2 RNA target.

## Supplementary Material

supinfo

## Data Availability

The data that support the findings of this study are available from the corresponding author upon reasonable request.
