# Supplementary material for: Gold Nanoparticle Enabled Localized Surface Plasmon Resonance on Unique Gold Nanomushroom Structures for On-Chip CRISPR-Cas13a Sensing
Source: Adv Mater Interfaces. Author manuscript; Available in PMC 2024 Jan 5. (PMC10121183; doi:10.1002/admi.202201261)
Supplement: supinfo [file NIHMS1853830-supplement-supinfo.pdf]

ADVANCED MATERIALS INTERFACES

Supporting Information

for *Adv. Mater. Interfaces,* DOI: 10.1002/admi.202201261

Gold Nanoparticle Enabled Localized Surface Plasmon Resonance on Unique Gold Nanomushroom Structures for On-Chip CRISPR-Cas13a Sensing

Jacob Waitkus, Yu Chang, Li Liu, Srinivasu Valagerahally Puttaswamy, Taerin Chung, Adrian M. Molina Vargas, Stephen J. Dollery, Mitchell R. O'Connell, Haogang Cai, Gregory J. Tobin, Nikhil Bhalla, and Ke Du**

Supporting Information

Gold Nanoparticle Enabled Localized Surface Plasmon Resonance on Unique Gold Nanomushroom Structures for On-Chip CRISPR-Cas13a Sensing

Jacob Waitkus, Yu Chang, Li Liu, Srinivasu Valagerahally Puttaswamy, Taerin Chung, Adrian M Molina Vargas, Stephen J Dollery, Mitchell R O'Connell, Haogang Cai, Gregory J Tobin, Nikhil Bhalla*, Ke Du*

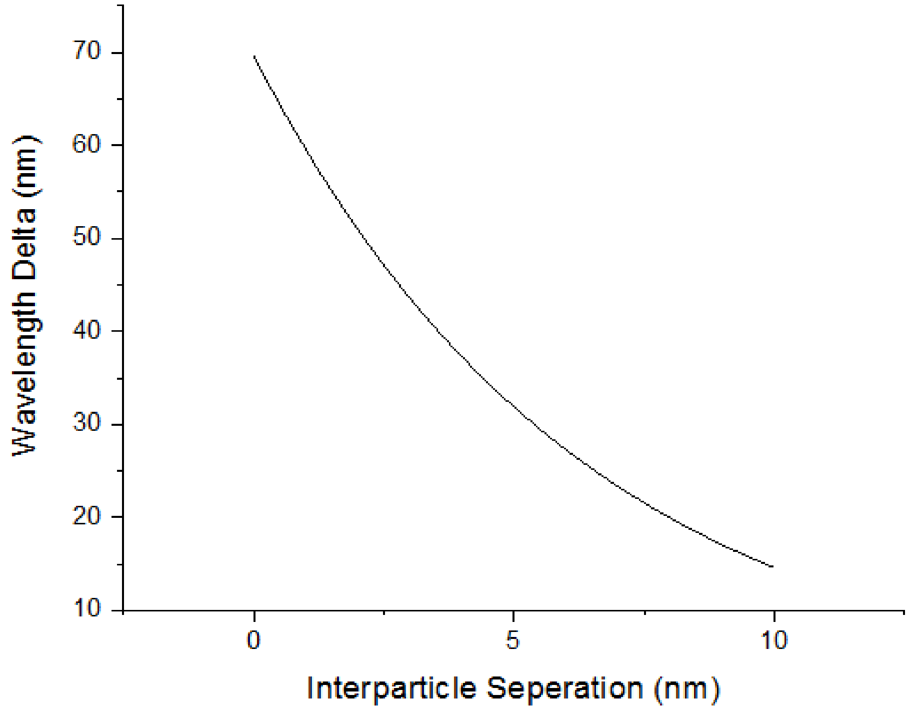

Figure S1. Graph obtained using the plasmonic ruler equation, adapted for AuNP-AuNM couplings to demonstrate the interparticle separation theorized for the experimental wavelength delta gathered.

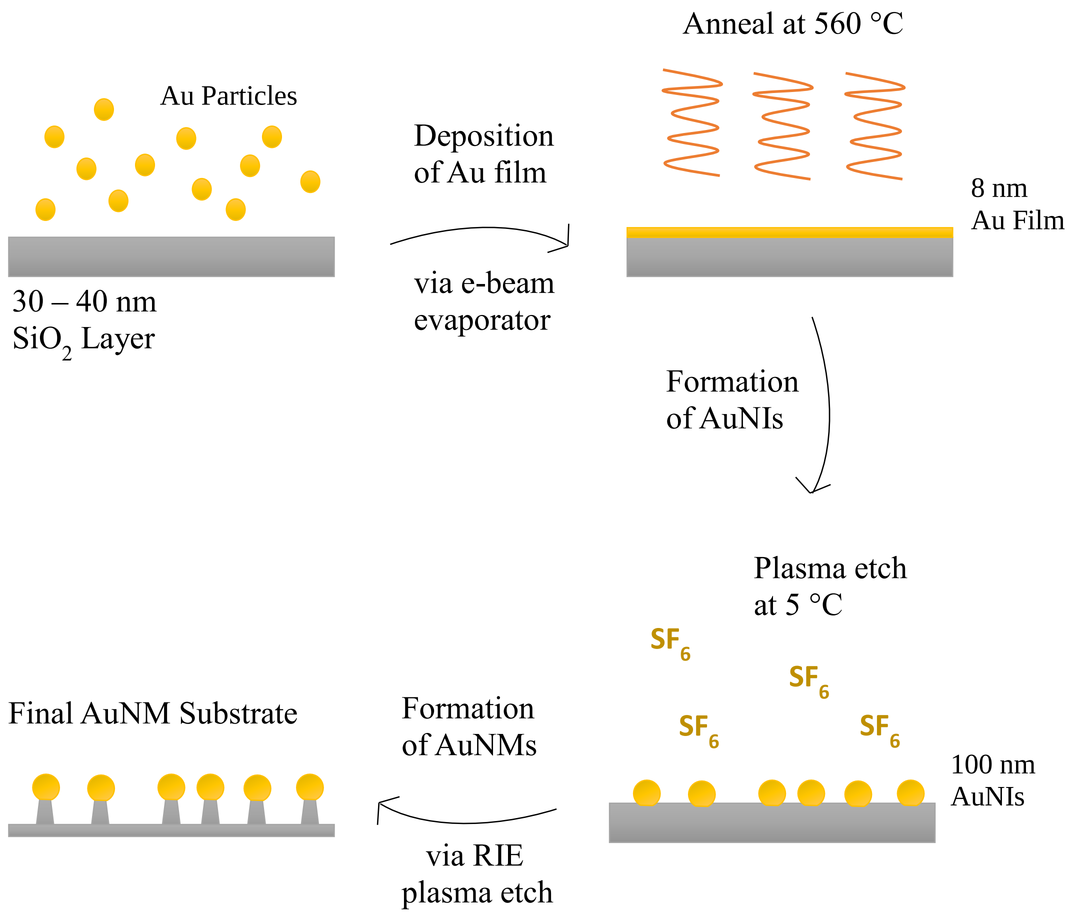

Figure S2. Detailed 2D image for the fabrication of the AuNM substrate[7].

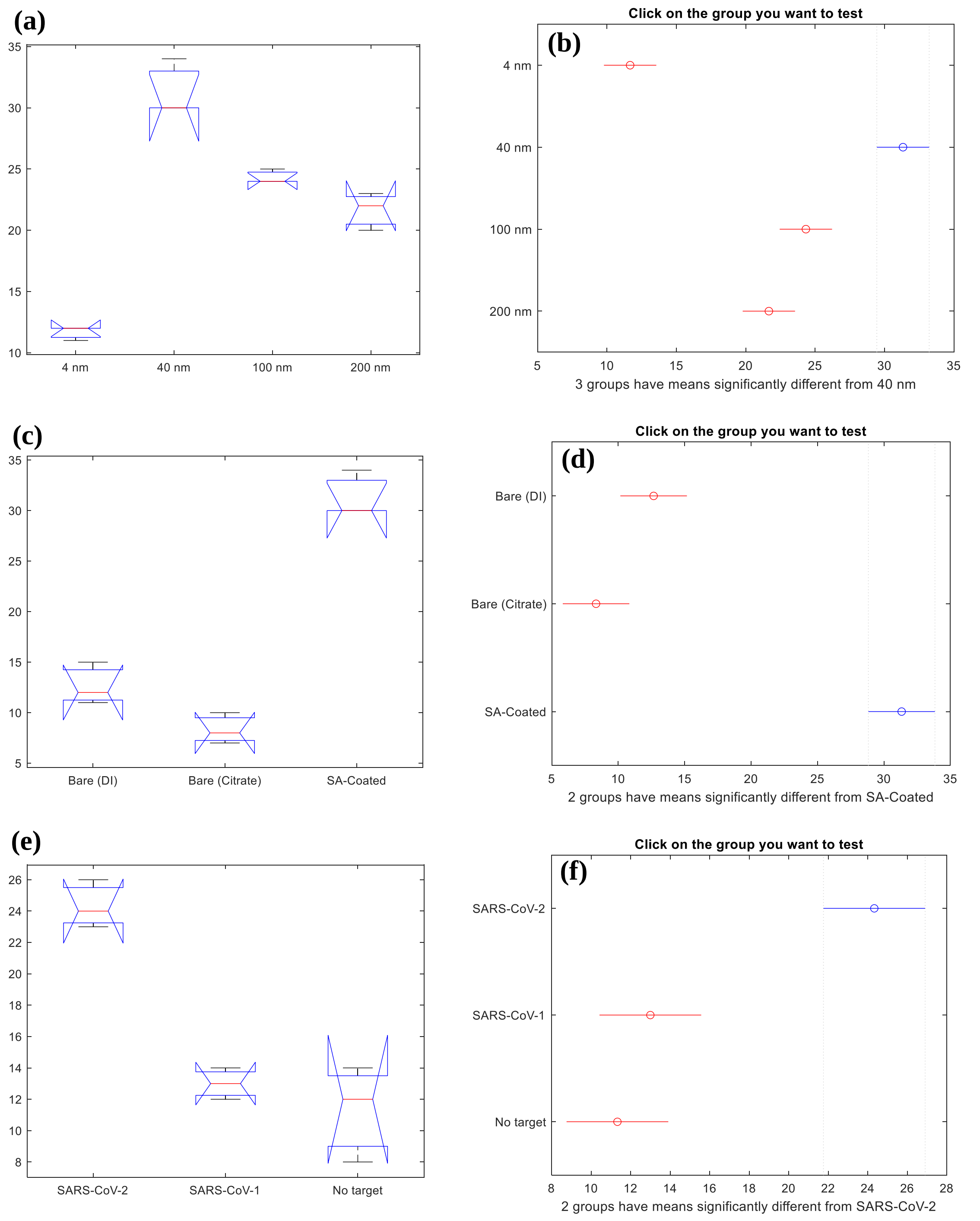

Figure S3. One-way ANOVA analysis for assessing the statistical significance for various experiments. Shown in both box plots and line graphs, the effects of (a, b) diameter and (c, d) AuNP coating, as well as the results from the (e, f) CRISPR-Cas13a testing. For each test the sample size (n) is equal to 3, and a P value less than 0.05 was used to measure statistical difference between experiments.

Table S1. Target RNA, crRNA, RNA reporter sequence

Name	Sequence	Sources
Fragment of plasmids pUC57-SARS-CoV-2	UUAUGUCCUU CCCUCAGUCA GCACCUCAUG GUGUAGUCUU CUUGCAUGUG ACUUAUGUCC CUGCACAAGA AAAGAACUUC ACAACUGCUC CUGCCAUUUG UCAUGAUGGA AAAGCACACU UUCCUCGUGA AGGUGUCUUU GUUUCAAAUG GCACACACUG GUUUGUAACA CAAAGGAAUU UUUAUGAACC ACAAAUCAUU ACUACAGACA ACACAUUUGU GUCUGGUAAC UGUGAUGUUG UAAUAGGAAU UGUCAACAAC ACAGUUUAUG AUCCUUUGCA ACCUGAAUUA GACUCAUUCA AGGAGGAGUU AGAUAAAUAU UUUAAGAAUC AUACAUCACC AGAUGUUGAU UUAGGUGACA UCUCUGGCAU UAAUGCUUCA GUUGUAAACA UUCAAAAAGA AAUUGACCGC CUCAAUGAGG UUGCCAAGAA UUUAAAUGAA UCUCUCAUCG AUCUCCAAGA ACUUGGAAAG UAUGAGCAGU AUAUAAAAUG GCCAUGGUAC AUUUGGCUAG GUUUUAUAGC UGGCUUGAUU GCCAUAGUAA UGGUGACAAU UAUGCUUUGC UGUAUGACCA GUUGCUGUAG UUGUCUCAAG GGCUGUUGUU CUUGUGGAUC CUGCUGCAAA UUUGAUGAAG ACGACUCUGA GCCAGUGCUC AAAGGAGUCA AAUUACAUUA CACAUAAACG AACUUAUGGA UUUGUUUAUG AGA	T7 run-off transcript
Fragment of plasmids pUC57-SARS-CoV-1	UUGUGUUUAA UGGCACUUCU UGGUUUAUUA CACAGAGGAA CUUCUUUUCU CCACAAAUAA UUACUACAGA CAAUACAUUU GUCUCAGGAA AUUGUGAUGU CGUUAUUGGC AUCAUUAACA ACACAGUUUA UGAUCCUCUG CAACCUGAGC UUGACUCAUU CAAAGAAGAG CUGGACAAGU ACUUCAAAAA UCAUACAUCA CCAGAUGUUG AUCUUGGCGA CAUUUCAGGC AUUAACGCUU CUGUCGUCAA CAUUCAAAAA GAAAUUGACC GCCUCAAUGA GGUCGCUAAA AAUUUAAAUG AAUCACUCAU UGACCUUCAA GAAUUGGGAA AAUAUGAGCA AUAUAUUAAA UGGCCUUGGU AUGUUUGGCU CGGCUUCAUU GCUGGACUAA UUGCCAUCGU CAUGGUUACA AUCUUGCUUU GUUGCAUGAC UAGUUGUUGC AGUUGCCUCA AGGGUGCAUG CUCUUGUGGU UCUUGCUGCA AGUUUGAUGA GGAUGACUCU GAGCCAGUUC UCAAGGGUGU CAAAUUACAU UACACAUAAA CGAACUUAUG GAUUUGUUUA UGAGAUUUUU UACUCUUAGA UCAAUUACUG CACAGCCAGU AAAAAUUGAC AAUGCUUCUC CUGCAAGUAC UGUUCAUGCU ACAGCAACGA UACCGCUACA	T7 run-off transcript
crRNA	GACCACCCCAAAAAUGAAGGGGACUAAAACgccagagaugucaccuaaau	IDT
RNA reporter	/56-FAM/rUrUrUrUrU/3BioTEG/	IDT

Table S2. One-way ANOVA analysis tables for experiments on (a) diameter, (b) coating, and (c) CRISPR-Cas13a testing.

(a) ANOVA Table

Source	SS	df	MS	F	Prob>F
Columns	597.583	3	199.194	95.61	1.31931e-06
Error	16.667	8	2.083		
Total	614.25	11			

(b) ANOVA Table

Source	SS	df	MS	F	Prob>F
Columns	762.889	2	381.444	127.15	1.22476e-05
Error	18	6	3		
Total	780.889	8			

(c) ANOVA Table

Source	SS	df	MS	F	Prob>F
Columns	300.222	2	150.111	35.55	0.0005
Error	25.333	6	4.222		
Total	325.556	8			
